# The Response of Flame-Retardant Polypropylene Composites After Long-Term Exposure to Corrosive Media

**DOI:** 10.3390/polym18202480

**Published:** 2026-10-11

**Authors:** Elena-Janina Vîlcea, Brîndușa Bălănucă, Mădălina Ioana Necolau, Manuela-Roxana Dijmărescu, Rodica-Mariana Ion, Maria-Cristina Toader

**Affiliations:** 1 Manufacturing Engineering Department, Faculty of Industrial Engineering and Robotics, National University of Science and Technology POLITEHNICA Bucharest, 060042 Bucharest, Romania; manuela.dijmarescu@upb.ro; 2 Department of Organic Chemistry “C. Nenitescu”, Faculty of Chemical Engineering and Biotechnology, National University of Science and Technology POLITEHNICA Bucharest, 011061 Bucharest, Romania; 3 Advanced Polymer Materials Group, National University of Science and Technology POLITEHNICA Bucharest, 011061 Bucharest, Romania; madalina.necolau@upb.ro; 4 Doctoral School of Engineering Sciences, Valahia University of Târgoviște, 13 Aleea Sinaia, 130004 Targoviste, Romania; rodica_ion2000@yahoo.co.uk; 5 National Institute for Research & Development for Chemistry and Petrochemistry, 202 Splaiul Independenței, 060021 Bucharest, Romania; 6 Academy of Romanian Scientists, 3 Ilfov Street, 030167 Bucharest, Romania; 7 Quality Engineering and Industrial Technologies Department, Faculty of Industrial Engineering and Robotics, National University of Science and Technology POLITEHNICA Bucharest, 060042 Bucharest, Romania; maria.dijmarescu@upb.ro

**Keywords:** polypropylene composites, phosphorus-based intumescent flame retardant, halogen-free flame retardant, aqueous and corrosive media, long-term exposure, absorption/degradation degree

## Abstract

This study evaluates the response of flame-retardant polypropylene composites after long-term exposure to aqueous and chemically aggressive media. Polypropylene (PP), used as a reference material, and PP-based composites containing 5, 10, 15, 20, and 25 wt.% of a phosphorus-based intumescent flame-retardant additive were immersed in water, NaCl solution, H_2_SO_4_ solution, and NaOH solution for up to 200 days. The material behavior was initially assessed by gravimetric measurements, based on the absorption/degradation degree calculated for early, mid, and late exposure intervals. This allowed the evolution of the material–medium interaction to be tracked not only by individual mass variations but also by average trends over the exposure period. The exposed systems were further investigated by thermogravimetric analysis (TGA/DTG) and dynamic mechanical analysis (DMA) to determine whether contact with the selected media modified the thermal degradation profile or the dynamic mechanical response of the composites. The results showed that the incorporated flame-retardant additive increases the interaction of PP with the liquid media, especially at intermediate and high additive contents. TGA/DTG and DMA confirmed that the thermal and viscoelastic behavior of the PP-based systems was maintained after long-term exposure, indicating that the observed medium-induced interactions did not result in substantial deterioration of their bulk thermo-mechanical properties.

## 1. Introduction

Polypropylene (PP) is used on a large scale because it offers a useful combination of low density, accessible cost, chemical resistance, and relatively simple processing. These characteristics explain its presence in packaging, automotive components, construction products, electrical applications, textiles, and consumer goods [1,2]. At the same time, the polymer has a major limitation for applications requiring fire safety. Its hydrocarbon structure favors ignition, melt dripping, and heat release, while protective char formation during combustion is limited [2,3,4,5]. For this reason, developing flame-retardant PP systems remains a practical and scientific challenge, especially when improving fire behavior must be achieved without losing processability or mechanical performance [3,4,5,6].

In recent years, attention has moved mainly toward halogen-free flame-retardant systems. Phosphorus-based, nitrogen-based, mineral, and hybrid intumescent formulations have been widely studied for PP and other polyolefins [3,4,6,7,8,9]. Phosphorus-containing compounds generally act through condensed-phase reactions, dehydration, and char formation, while nitrogen-containing components may contribute by releasing non-combustible gases and expanding the protective layer [4,7,8,9]. Mineral and layered additives, such as magnesium hydroxide, molecular sieves, montmorillonite, zeolites, and related silicate phases, may further improve the barrier effect by increasing the stability and compactness of the residue [10,11,12,13,14].

However, the efficiency of a flame-retardant formulation cannot be evaluated only through fire-performance indicators. A material that improves the limiting oxygen index, UL-94 classification, or cone-calorimetry response is not necessarily suitable if the additive loading is too high, if the matrix becomes weaker, or if the interface becomes sensitive to water and environmental exposure [2,3,5,7,9]. Several studies have therefore focused on surface modification, layered distribution, or synergistic additive combinations in order to improve compatibility with the PP matrix and to reduce the loss of mechanical performance after exposure [15,16,17,18,19,20,21,22]. From this point of view, flame-retardant PP composites have to be analyzed not only as materials with improved fire behavior but also as systems that must maintain their structure and properties during service.

Interaction with liquid media is important for this type of material because water or chemical solutions can enter the composite through diffusion, absorption, and interfacial pathways. Free volume, voids, filler/matrix adhesion, and the possible extraction of low-molecular-weight or soluble fractions may influence the process [23,24,25,26,27,28,29]. In polymer composites, moisture uptake may follow Fickian or non-Fickian behavior and can lead to swelling, plasticization, interfacial stress, mass loss, or changes in thermo-mechanical response, depending on the matrix and the filler system [23,24,25,26,27,28,29]. In PP, the matrix’s hydrophobic character limits direct water uptake, but additives or fillers can create additional regions where aqueous media can interact with the material [23,25,27,28,29].

Therefore, a positive mass variation measured after immersion should not be interpreted immediately as degradation. In many cases, it may indicate liquid uptake, temporary sorption, interfacial transport, or absorption–desorption processes. A more reliable interpretation requires correlating the gravimetric response with thermal and dynamic mechanical data. If the main degradation profile and the viscoelastic response remain comparable after exposure, the mass variation can be discussed mainly as a physical interaction with the media.

Water resistance is particularly relevant for intumescent flame-retardant PP systems because some phosphorus–nitrogen formulations may contain components that are sensitive to moisture, migration, or interfacial weakening. Recent studies on modified ammonium polyphosphate and coated intumescent additives treat water resistance, flame-retardant performance, and mechanical properties as related requirements, not as independent characteristics [21,30]. Therefore, for PP systems containing phosphorus–nitrogen additives, the exposure environment may influence both the additive-rich regions and the matrix-additive interface.

Studies on thermoplastic composites exposed to water, salt water, acidic solutions, and alkaline solutions show that aging can modify water uptake, mechanical response, thermal behavior, and even flammability. The intensity and direction of these changes depend on the polymer matrix, the reinforcement or additive, and the chemistry of the exposure medium [31]. So, the durability of flame-retardant PP composites should be evaluated through a combined approach, especially when the material is intended for conditions where contact with aqueous or chemically aggressive media is possible.

The additive used in this study belongs to the group of phosphorus–nitrogen intumescent flame-retardant additives previously investigated in PP-based systems. The available literature on flame-retardant PP composites containing this type of additive is more limited than the general literature on flame-retardant PP. Earlier work reported the use of this additive in PP and analyzed its influence on polymer structure and properties [32]. Other studies considered PP-based composite formulations with 5–25 wt.% additive and reported composition-dependent mechanical behavior [33]. These results confirm the interest of flame-retardant PP composites as halogen-free systems, but they do not provide sufficient information regarding their response after prolonged contact with aqueous, saline, acidic or alkaline media.

In the present study, PP and flame-retardant PP composites containing 5, 10, 15, 20, and 25 wt.% of the phosphorus–nitrogen intumescent flame-retardant additive are analyzed after long-term immersion in water, NaCl, H_2_SO_4_, and NaOH solutions. The gravimetric response is followed over early, mid, and late exposure intervals, up to 200 days. After immersion, the exposed systems are further investigated by thermogravimetric analysis (TGA/DTG) and dynamic mechanical analysis (DMA).

This research aims to determine how the additive content influences apparent mass uptake or mass loss in the selected media and whether the main thermal degradation behavior and dynamic mechanical response of the PP-based systems are preserved after prolonged exposure.

## 2. Materials and Methods

### 2.1. Materials

The materials considered in this study were polypropylene-based systems containing different amounts of a phosphorus-based intumescent flame-retardant additive. The polymer matrix was isotactic polypropylene (PP). The PP-based formulation without this additive served as the reference material, while the modified systems were analyzed to determine how the additive content influenced the material behavior after long-term exposure to liquid media.

The initial PP-based material also contained calcium carbonate and a thermally stable green pigment. These components were already present in the polypropylene plate and were not varied in the present experimental program. Consequently, the main compositional variable followed in this study was the flame-retardant additive content.

The additive used in this study was a commercial halogen-free intumescent flame-retardant additive, known as Reogard 2000 ^®^ (Great Lakes Chemical Corporation, Indianapolis, IN, USA) [33]. In the context of the present paper, the additive was not investigated through direct fire-testing methods, but through its influence on the long-term response of the PP-based systems after contact with aqueous and chemically aggressive environments.

Five additive concentrations were considered: 5, 10, 15, 20, and 25 wt.% of the phosphorus-based intumescent flame-retardant additive. The investigated materials were coded as PP (the initial PP-based material), PP+5%R, PP+10%R, PP+15%R, PP+20%R, and PP+25%R, where R denotes the additive, and PP-R refers to the flame-retardant composite systems. This notation was used throughout the paper to allow a direct comparison between polypropylene material and the additive-containing composites.

Water, sodium chloride solution (NaCl, 3.5%), sulfuric acid solution (H_2_SO_4_, 1 M), and sodium hydroxide solution (NaOH, 1 M) were used as exposure media for the long-term immersion study. These media were selected in order to compare the response of the materials investigated under neutral aqueous, saline, acidic, and alkaline conditions.

### 2.2. Methods

#### 2.2.1. Composite Fabrication

The flame-retardant PP composites were prepared to obtain plate-type specimens suitable for the long-term immersion study and subsequent thermal and dynamic mechanical investigations. For each composition, the required amount of the additive was introduced into the PP-based material according to the additive concentration established in Section 2.1.

The flame-retardant PP composites were prepared based on a previously reported protocol for PP systems containing the same phosphorus-based intumescent flame-retardant additive [34]. The PP-based material was first dry-blended with the required amount of additive. The mixtures were subsequently melt-mixed at 180–190 °C to obtain a uniform distribution of the additive within the polymer matrix. After mixing, the materials were shaped into plates by compression molding. The same preparation procedure was applied for all compositions, with the additive content varied from 5 to 25 wt.%.

After fabrication, the plates were conditioned at room temperature for 48 h before cutting and testing to reduce the influence of short-term moisture variation and residual stress generated during processing. Specimens with comparable geometry were then cut from the obtained plates and used for the immersion tests, TGA/DTG analysis, and DMA measurements.

**Figure 1:**
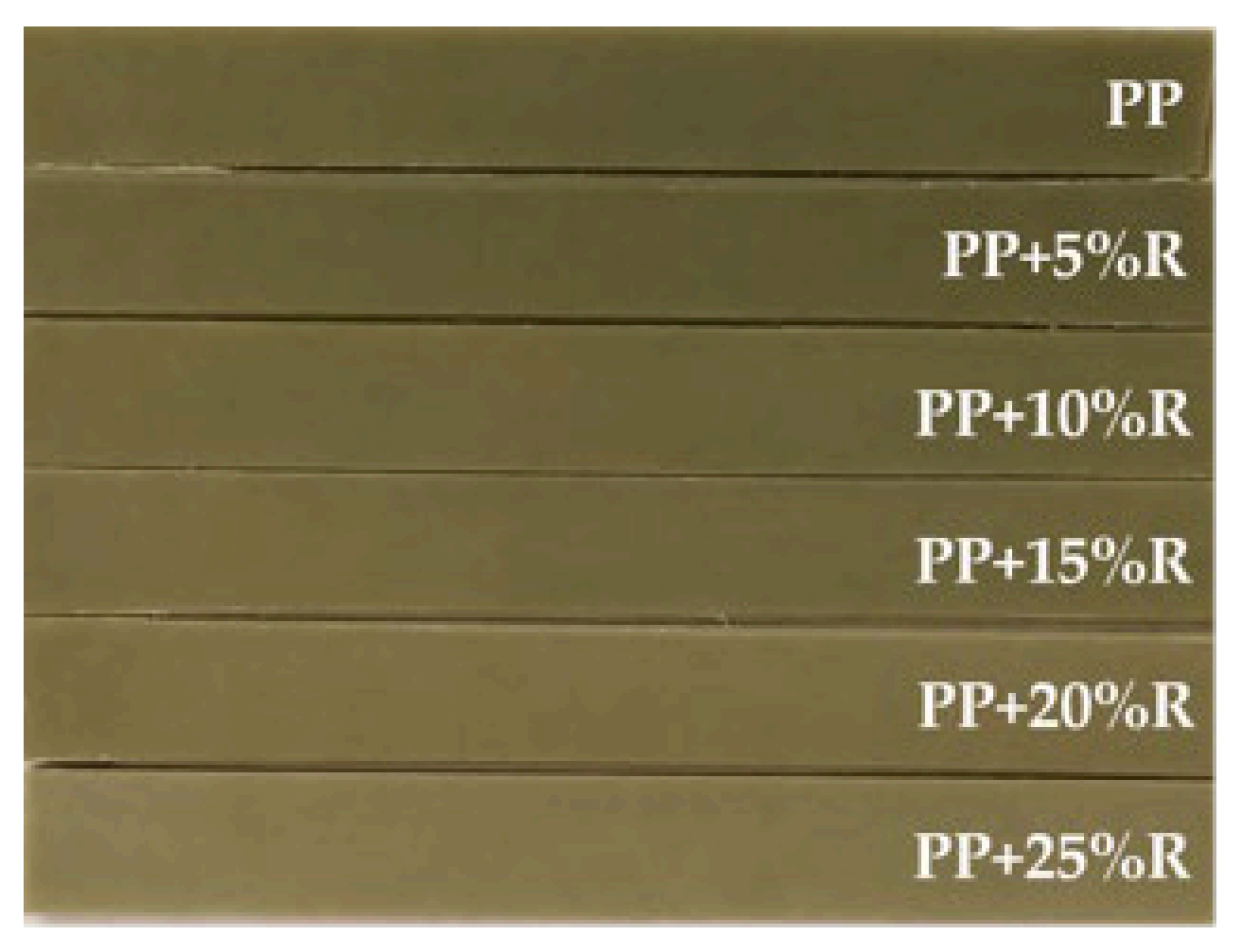
Representative image of the prepared flame-retardant PP plate-type strips from which the specimens used for the immersion tests were cut, coded according to the compositions defined in Section 2.1.

Figure 1 presents a representative image of the prepared plate-type strips from which the specimens used for the immersion tests were subsequently cut. The strips show the characteristic green color of the PP-based material, associated with the thermally stable pigment present in the initial formulation.

The specimens maintained the characteristic green color of the PP-based formulation, but differences in shade can be observed between the investigated compositions. Depending on the flame-retardant additive content, some samples show darker or more olive-green tones. As color was not quantitatively analyzed in this study, these differences were considered only as a qualitative characteristic of the prepared materials.

#### 2.2.2. Characterization of the PP-R Composites

*Long-term absorption behavior in aqueous and corrosive media*. Several degradative media were evaluated for PP-R composites: water, NaCl solution (3.5%), acid (H_2_SO_4_ solution, 1 M), and alkaline medium (NaOH solution, 1 M). Rectangular specimens cut from the samples presented in Figure 1 were prepared, with approximate dimensions of 10 mm long, 12.5 mm wide, and 3.2 mm thick.

PP-R composites were exactly weighed (m_i_) and held in each medium at room temperature. At a certain incubation time (from 7 to 200 days), each PP-R specimen was extracted, carefully dried with filter paper, ensuring the removal of liquid from all surfaces, and then reweighed (m_f_). The PP-R samples were maintained under static immersion conditions for the entire exposure period (contact with the same immersion medium throughout 200 days).

The obtained mass values were used to calculate the absorption/degradation degree (AD) using Equation (1).

(1) $$\mathrm{AD}\;\left(\%\right)=\frac{\left(\mathrm{mi}-\mathrm{mf}\right)}{\mathrm{mi}}\;\times \;100$$

For comparative interpretation of the long-term gravimetric response, the experimental period was divided into three characteristic intervals: early (7–28 days), mid (35–100 days), and late (120–200 days). The reported values for each exposure interval represent the arithmetic mean of the AD values obtained at the individual immersion times included in that interval and, consequently, describe the average temporal response over the corresponding exposure period:

AD_Early_ = mean [AD (7 days), AD (14 days), AD (21 days), AD (28 days)].

The early exposure interval was calculated excluding time zero, which was considered a reference state.

AD_Mid_ = mean [AD (35 days), AD (40 days), AD (50 days), AD (60 days), AD (70 days), AD (80 days), AD (90 days), AD (100 days)].

AD_Late_ = mean [AD (120 days), AD (140 days), AD (170 days), AD (200 days)].

*Thermogravimetric analysis (TGA)* was performed using Netzsch TG 209 F1 Libra equipment (NETZSCH-Gerätebau GmbH, Selb, Germany), in the temperature range from ambient to 800 °C, under a nitrogen atmosphere with a heating rate of 10 °C/min.

Thermograms were recorded under the same conditions for both the initial materials and those obtained after the long-term absorption test.

*Dynamic mechanical analysis (DMA)*. Thermo-mechanical properties were investigated through DMA using TRITEC 2000 B equipment (Triton Technology Ltd., Loughborough, UK). The program parameters used were set as follows: temperature between −25 and 140 °C, heating rate of 4 °C/min, in single cantilever bending mode, using a frequency of 1 Hz. DMA was performed for both initial materials and those exposed to the long-term absorption test under the same analysis conditions.

## 3. Results and Discussion

The results obtained in the present study are discussed in relation to the interaction between the flame-retardant PP composites and the selected exposure media. Because the investigated materials were immersed for a long period in water, NaCl, H_2_SO_4_, and NaOH solutions, the interpretation was not limited to a simple comparison of mass variation values but also considered the evolution of the material response with exposure time and additive content. Assessing flame-retardant PP composites’ long-term behavior in chemically different liquid environments is crucial to determining how durable they are under practical service circumstances. For moisture-induced effects, water serves as a baseline; saline, acidic, and alkaline environments are represented by NaCl, H_2_SO_4_, and NaOH solutions, respectively. The systematic exposure to these media reveals whether adding more of the flame-retardant additive weakens the PP matrix’s inherent durability or, on the other hand, results in quantifiable medium–material interactions without significantly altering the composites’ bulk thermal and viscoelastic properties.

### 3.1. Long-Term Absorption Behavior in Aqueous and Corrosive Media

A long-term immersion study, extending up to 200 days, was conducted to evaluate the absorption and degradation behavior of PP-R composites and to assess their resistance under various corrosive environments: water, saline, acidic, and alkaline media, related to their additive content.

The experimental dataset contains the gravimetric response of each investigated PP-R formulation at 16 successive immersion times in each exposure medium. Due to the very large number of experimental time points according to the testing duration, the resulting datasets for each corrosive medium were systematically divided into three characteristic exposure intervals: early, mid, and late, in order to facilitate a clearer interpretation of the temporal evolution of material behavior.

Specifically, the early interval (0–28 days) represents the initial interaction between the composites and the corrosive medium (time 0 was excluded, being the reference state), the mid interval (35–100 days) reflects the transition toward a more advanced swelling or degradation process, and the late interval (120–200 days) represents long-term exposure effects. The mean absorption/degradation degree (AD) calculated for each interval was subsequently used for comparative analysis among the investigated composite systems.

#### 3.1.1. Water Exposure

**Figure 2:**
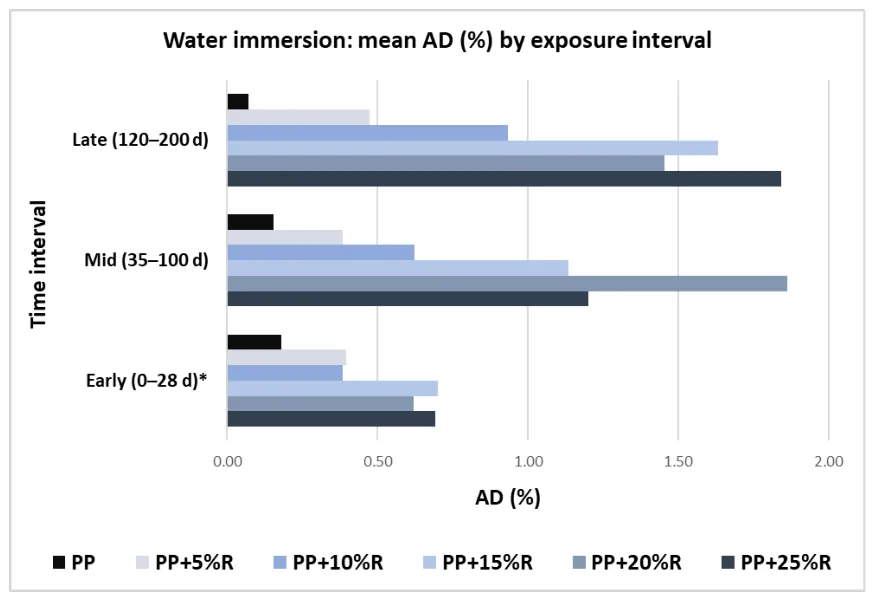
PP-R behavior in water. * Day 0 was excluded from the calculation of the mean AD value, as it represents the reference state.

*Early exposure interval (0–28 days)*. All investigated systems exhibit positive absorption/swelling degree values, indicating a rapid but decreased water uptake (Figure 2). Reference PP shows very low mean AD values (0.119–0.238, with a mean AD of 0.179%), consistent with its inherently hydrophobic nature and limited interaction with water.

Additive-containing systems display moderately higher AD values, even at low additive contents, reflecting an increased affinity for water and facilitated initial diffusion within the polymer matrix. This behavior indicates a surface- and diffusion-controlled response in an aqueous medium. The relatively small differences among the modified systems (e.g., 0.699% for 15% additive content; 0.691% for 25% additive content) suggest that early-stage absorption is governed primarily by interfacial phenomena.

*Mid exposure interval (35–100 days)*. This interval represents the most sensitive stage of the immersion test, corresponding to the transition from initial water uptake to a more developed interaction between the polymer matrix and the aqueous environment. During this exposure interval, the differences between reference PP and additive-loaded composites become more pronounced. While PP maintains low and constant AD values (0.154%), the modified systems exhibit increased mean AD values with a clear dependence on additive content (0.385% for PP+5%R; 0.622% for PP+10%R; 1.136% for PP+15%R; 1.861% for PP+20%R; 1.202% for PP+25%R).

Some isolated negative AD values (mass loss) are observed for PP in the raw experimental data at specific time points (70–90 days of immersion), but these events do not accumulate over time. Their absence in the averaged interval data indicates that these mass fluctuations are not associated with progressive degradation, but rather with a minor removal of low-molecular-weight fractions.

*Late exposure interval (120–200 days)*. Reference PP exhibits the same mean AD values close to zero, confirming its long-term stability in water. Additive-modified composites, by contrast, retain elevated but stable AD values, reflecting constant but non-destructive water uptake behavior.

It can be assumed that, despite the extended immersion time, no degradation trend was observed for the modified systems, indicating that the flame-retardant additive increased hydrophilicity without causing long-term degradation of the polymer matrix.

The different time-dependent AD trends observed for neat PP and PP-R composites can be associated with the additional interactions introduced by the Reogard filler and the resulting polymer–filler interfaces. Neat PP exhibits intrinsically low water uptake, while an increased water absorption with increasing filler content has been reported for filled PP systems and is associated, among other factors, with interfacial regions between the filler and the polymer matrix [35]. More generally, matrix–filler interfaces have been identified as possible additional pathways for moisture transport in particle-filled polymer composites, alongside diffusion through the polymer matrix [25]. Accordingly, the generally higher and, for several PP-R composites, progressively increasing AD values may reflect an enhanced contribution of the filler-containing structure and polymer–filler interfaces to medium penetration and retention.

#### 3.1.2. NaCl Solution Exposure

*Early exposure interval (0–28 days)*. All systems exhibit positive mean AD values, indicating initial mass uptake upon immersion in the NaCl solution. Reference PP shows minimal interaction with the saline medium, confirming its hydrophobic and chemically inert character. PP-R systems display moderately increased AD values, reflecting a greater affinity for the aqueous saline environment.

However, differences among the modified composites are limited at this stage, suggesting early behavior associated with surface wetting and superficial diffusion rather than extensive structural changes or degradation.

**Figure 3:**
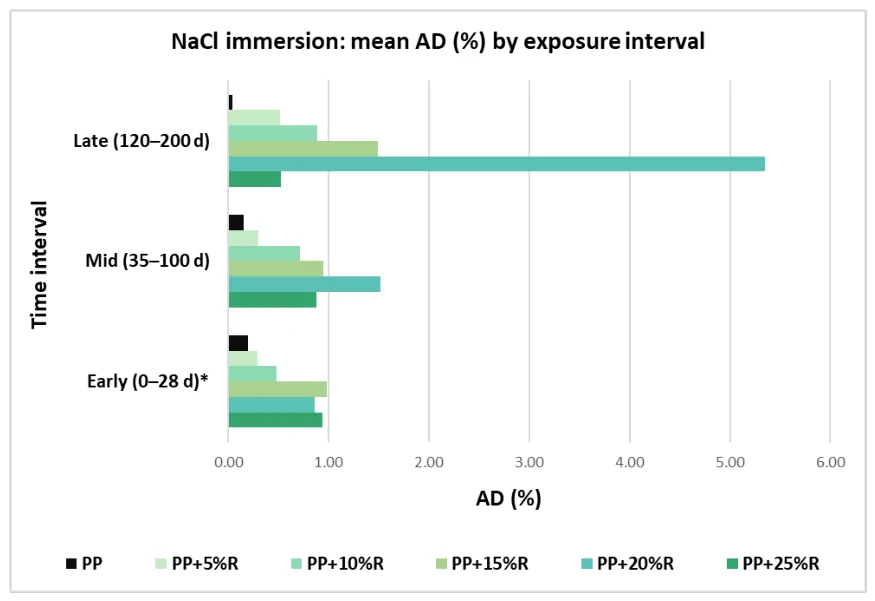
PP-R behavior in NaCl solution. * Day 0 was excluded from the calculation of the mean AD value, as it represents the reference state.

*Mid exposure interval (35–100 days)*. A clearer differentiation of the composite systems becomes evident during this mid interval, as shown in Figure 3. While PP maintains very low AD values, the additive-containing materials show a progressively higher AD with the increase of the additive content, indicating that water and ionic species may penetrate deeper into accessible regions of the polymer matrix.

A more pronounced absorption is observed for PP+20%R system, which stands out, but this behavior should not be interpreted as the onset of degradation. Examination of the raw gravimetric data indicates that this apparent anomaly originates from a localized increase between specific time points (notably around 90–100 days), rather than from a continuous or cumulative mass change. Such a transient deviation is consistent with temporary microstructural rearrangements, salt-assisted swelling, or localized osmotic effects, which can enhance fluid uptake without compromising the integrity of the polymer matrix [24]. Moreover, water or aqueous media diffusion and sorption in polymeric materials are governed by a range of physical mechanisms, often described by diffusion models, and do not inherently imply chemical degradation of the polymer network when absorption stabilizes at long times. The observed absorption behavior aligns with the established understanding of water diffusion in polymers, where moisture uptake proceeds via diffusion-controlled mechanisms, and long-term stabilization at positive or low AD values does not indicate chemical degradation. Comprehensive reviews show that water diffusion phenomena in polymers can be described by Fickian or non-Fickian processes without necessitating irreversible breakdown of the polymer network [23].

*Late exposure interval (120–200 days)*. Reference PP remains essentially unaffected by prolonged NaCl exposure, maintaining AD values close to zero. The additive-loaded composites retain elevated but stable mean AD values, indicating sustained fluid uptake rather than progressive degradation. Importantly, PP+20%R composite continues to exhibit high AD values throughout this late exposure, confirming that the pronounced response observed in the mid interval is not transient degradation but part of a consistent long-term behavior.

For the PP+25%R system, the mean AD values decrease progressively from the early to the late exposure interval, indicating a gradual net mass loss with increasing immersion time; however, the magnitude of this decrease remains limited and does not suggest accelerated degradation under NaCl exposure.

#### 3.1.3. H_2_SO_4_ Solution Exposure

*Early exposure interval (0–28 days)*. All systems exhibit positive AD values, indicating initial mass uptake in the acidic medium. Reference PP shows a limited response, while additive-modified systems display moderately higher AD values, suggesting increased interaction with the H_2_SO_4_ solution without evidence of rapid degradation. The differences between the additive-based composites remain relatively small at this stage, indicating that early behavior is dominated by surface effects.

*Mid exposure interval (35–100 days)*. A clear separation between reference PP and additive-containing systems becomes apparent. The mean AD values increase with the additive content (from 0.323% for PP+5%R to 0.961% for PP+15%R or 0.943% for PP+20%R), reflecting enhanced accessibility of the acidic medium within the composite structure, compared with the reference PP, which indicates a clear transition from limited absorption to net mass loss at longer immersion times in H_2_SO_4_, registering the mean AD of 0.056%.

Despite the aggressive nature of H_2_SO_4_, no abrupt decreases in AD were observed for the PP-R composites, indicating the absence of accelerated chemical degradation during this interval. The progressive increase in mean AD values observed from reference PP to additive-modified systems is consistent with literature reports on particle-filled polymer composites, where higher contents of hydrophilic fillers enhance the accessibility of acidic media through interfacial diffusion pathways. Moreover, at high filler or additive contents, moisture uptake may reach a saturation limit or even decrease due to microstructural densification and reduced free volume, despite increased hydrophilic fraction. This may explain the lower mean AD values for PP+25%R compared with other additive-based systems, since no negative AD was calculated for this system [25].

In this context, the non-monotonic behavior, with maximum AD values recorded at intermediate additive contents (15–20%), is consistent with classical polymer physics concepts describing an optimum additive concentration due to microstructural and mobility constraints [36].

*Late exposure interval (120–200 days)*. The additive-containing systems retain elevated and stable AD values, confirming sustained but non-destructive interaction with the acidic environment. Reference PP continues to show AD values close to zero throughout long-term exposure, with a negative mass at certain experimental points (90 to 140 days), clearly indicating progressive mass loss under prolonged H_2_SO_4_ exposure. The absence of negative AD values for PP-R composites demonstrates that long-term immersion in H_2_SO_4_ does not lead to their degradation.

**Figure 4:**
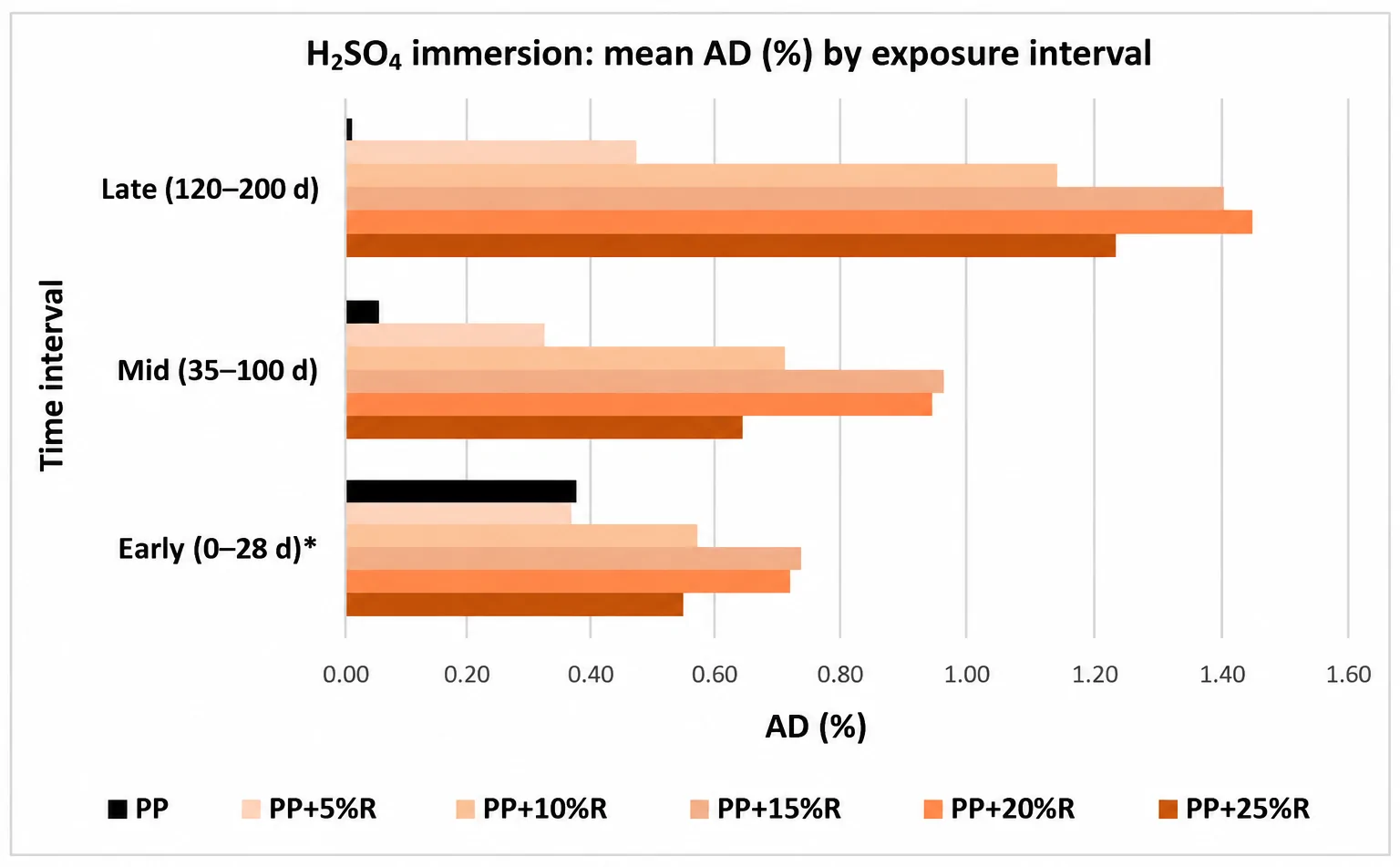
PP-R behavior in H_2_SO_4_ solution. * Day 0 was excluded from the calculation of the mean AD value, as it represents the reference state.

Overall, the AD trends observed across all exposure intervals (Figure 4) indicate that the incorporation of the phosphorus-based intumescent flame-retardant additive increases the interaction with the acidic medium without compromising the long-term resistance of the polypropylene matrix.

#### 3.1.4. NaOH Solution Exposure

**Figure 5:**
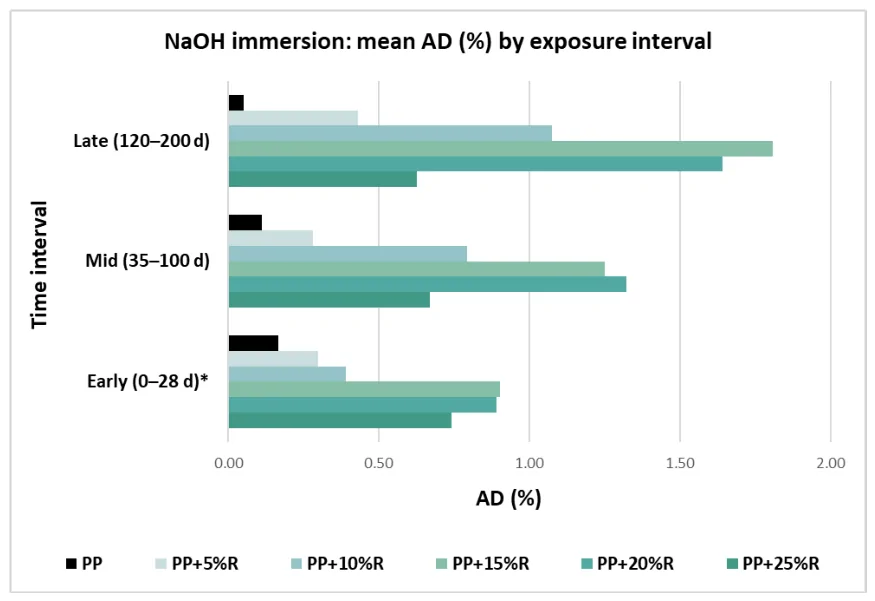
PP-R behavior in NaOH solution. * Day 0 was excluded from the calculation of the mean AD value, as it represents the reference state.

*Early exposure interval (0–28 days)*. All studied systems exhibit positive AD values, indicating initial mass uptake upon contact with the alkaline medium (Figure 5). Reference PP shows a limited response with a mean AD of 0.165%, while additive-loaded systems display higher AD values (0.2971% for PP+5%R; 0.392% for PP+10%R; 0.904% for PP+15%R; 0.890% for PP+20%R; 0.741% for PP+25%R), reflecting increased interaction with the NaOH solution without evidence of any degradation, suggesting an early behavior associated to surface interactions.

*Mid exposure interval (35–100 days)*. A clear differentiation between reference PP and additive-containing systems becomes evident. Based on raw data, reference PP undergoes alternating episodes of net mass loss followed by partial fluid uptake, resulting in a non-monotonic AD profile that reflects repeated loss-absorption cycles rather than stable sorption behavior in the alkaline medium.

For the composites, the mean AD values increase with the additive content, indicating enhanced accessibility of the alkaline medium within the composite structure.

However, when considering the full-time AD datasets, additive-containing composites also exhibit qualitatively similar non-monotonic behavior like the PP matrix, with alternating mass loss and fluid uptake events; the primary effect of the incorporation of the flame-retardant additive is an increase in the magnitude of these interactions rather than a change in the underlying mechanism.

*Late exposure interval (120–200 days)*. Composites containing the flame-retardant additive retain elevated and stable AD values, confirming sustained but non-destructive interaction with the alkaline environment.

Notably, the PP+25%R system exhibits AD values in this exposure interval that are comparable to those observed during the early and mid exposure stages, indicating a consistent and stabilized interaction with the alkaline medium over the entire immersion period. In contrast, reference PP continues to exhibit mass loss, indicating that the loss–uptake cycles observed in the mid stage persist under prolonged alkaline exposure.

The absence of negative AD values indicates that long-term NaOH immersion does not lead to significant material loss or chemical degradation of the composites.

The gravimetric response is also strongly medium-dependent, as particularly illustrated by PP+25%R, for which AD increases with prolonged exposure to water and H_2_SO_4_ but decreases in NaCl and NaOH. Moisture sensitivity and additive exudation have been reported for PP composites containing phosphorous–nitrogen intumescent additives based on melamine phosphate and pentaerythritol derivatives [37]. Therefore, differences in pH and ionic composition of the immersion media may influence the balance between medium uptake/retention and the mobility of additive-related species. The opposite AD observed for PP+25%R may thus reflect different balances between these processes. However, since gravimetric measurements cannot distinguish their individual contributions, this interpretation should be regarded as a plausible explanation rather than a demonstrated chemical mechanism.

Across all investigated environments (water, NaCl, H_2_SO_4_, and NaOH), the PP-R composites exhibit stable long-term behavior, characterized predominantly by limited and medium-dependent mass variations rather than by a clear trend of progressive mass loss. Although the incorporation of the flame-retardant additive enhances the interaction with the surrounding media, it does not appear to compromise the overall stability of the polypropylene matrix under prolonged exposure.

It should be noted that the experiments were conducted under static conditions (prolonged contact with non-renewed media). Consequently, the chemistry of the exposure media may have evolved during prolonged contact with the PP-R specimens. Possible changes in pH or concentration and/or accumulation of species released from the material may therefore have contributed to the observed long-term gravimetric response. Since the evolution of the exposure medium chemistry was not characterized during immersion, these contributions cannot be quantitatively distinguished from other processes affecting the measured net mass variation.

### 3.2. Thermogravimetric Analysis (TGA)

**Figure 6:**
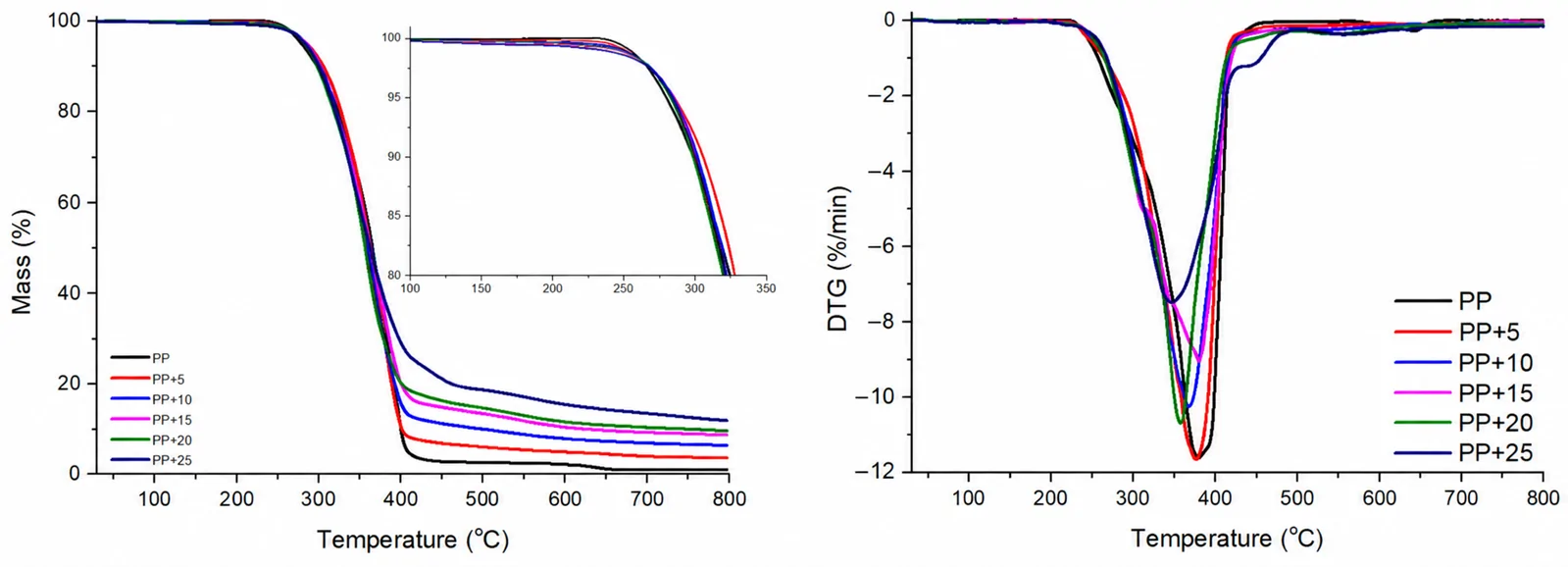
TGA (**left**) and DTG (**right**) curves of the initial PP-R composites (before degradation study).

First, the initial reference PP and corresponding composites were investigated in order to establish their thermal resistance. All investigated systems exhibit a single main degradation step, characteristic of polypropylene-based materials, indicating a similar thermal decomposition mechanism for reference PP and additive-loaded composites, as depicted in Figure 6(left).

The onset and temperature range of the main degradation process show only minor variations with increasing additive content, suggesting that the incorporation of the flame-retardant additive does not significantly affect the thermal stability of the PP matrix. An increase in residual mass at high temperatures is observed with increasing additive content, indicating the contribution of the phosphorus-based intumescent flame-retardant additive to a thermally stable residue formation.

The DTG curves (Figure 6(right)) display a single dominant degradation peak for all compositions, confirming a one-step degradation process prior to environmental exposure. The position of the maximum degradation rate (T_max_) remains comparable for all studied systems, with only slight shifts observed for higher additive contents.

Notably, the PP+15%R system exhibits a T_max_ value comparable to those of reference PP and PP+5%R, indicating that the incorporation of 15% Reogard does not produce a notable shift in the temperature of maximum degradation rate under the investigated conditions. Overall, the variation of T_max_ with Reogard content is non-monotonic, indicating that the thermal response does not follow a simple concentration-dependent trend. Variation in thermal degradation behavior following the incorporation of additives has been reported for PP-based composite systems [38].

Similar behavior has been reported in polypropylene composites with inorganic fillers, where the thermal stability and degradation profiles measured by TGA depend on filler content and modification, reflecting the influence of filler dispersion and matrix–filler interactions on degradation kinetics rather than a simple shift in degradation temperature [38].

Minor variations in peak intensity reflect differences in degradation kinetics, while the overall similarity of the DTG profiles (Figure 6(right)) indicates that the additive used in this study does not introduce additional degradation mechanisms.

After long-term exposure to corrosive media (water, NaCl, NaOH, and H_2_SO_4_), the TGA and DTG profiles remain almost comparable to those registered for the initial materials, preserving the single-step degradation mechanism.

Thermogravimetric analysis of the non-incubated systems shows that all PP-R composites undergo thermal degradation within a similar temperature range, with TGA mass loss starting below 250 °C and completing around 400 °C, while DTG reveals that T_max_ values are dependent on the additive content. Thus, at intermediate additive contents (15%), the degradation behavior remains comparable to reference PP, whereas higher additive loadings lead to slightly lower T_max_ values, reflecting changes in degradation kinetics.

After long-term exposure to different corrosive media, the DTG maxima of both reference PP and additive-containing composites shift toward higher temperatures, clustering around ~380–400 °C, with the exact position depending on the exposure medium and filler content.

**Figure A1:**
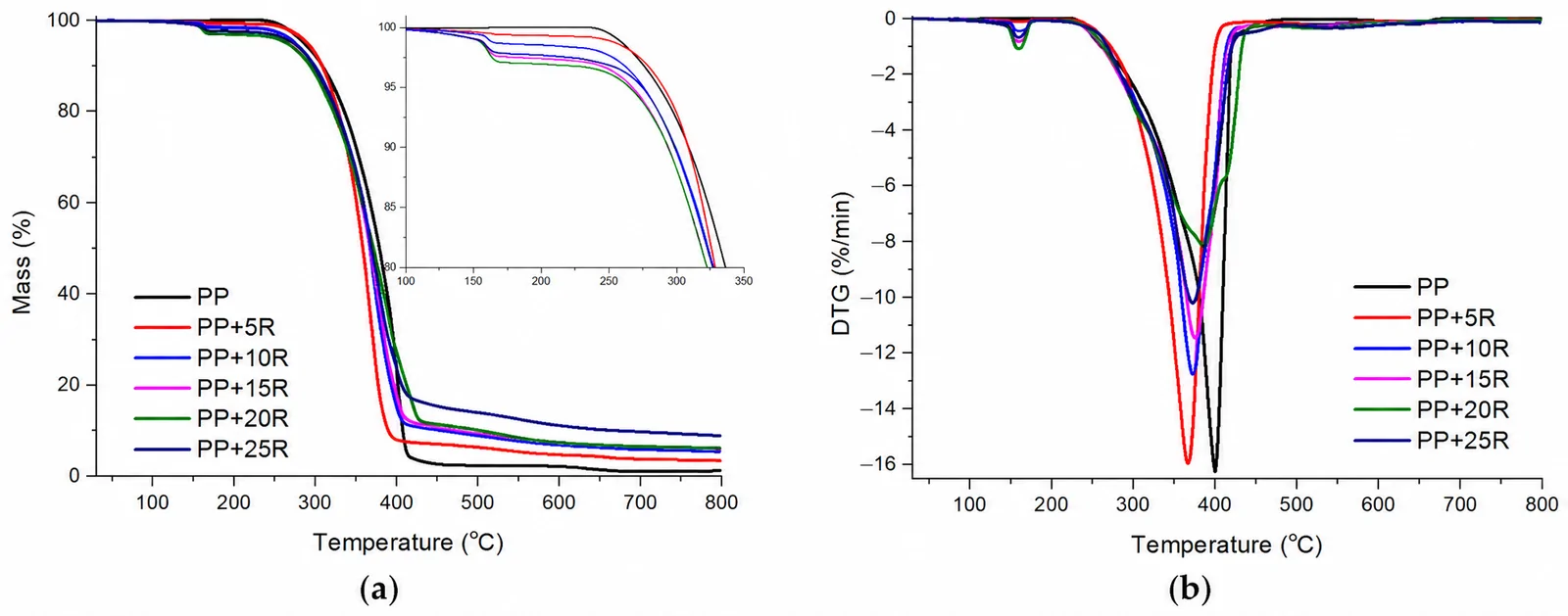
TGA (**a**) and DTG (**b**) curves of the PP-R composites after long-term water absorption.

**Figure A2:**
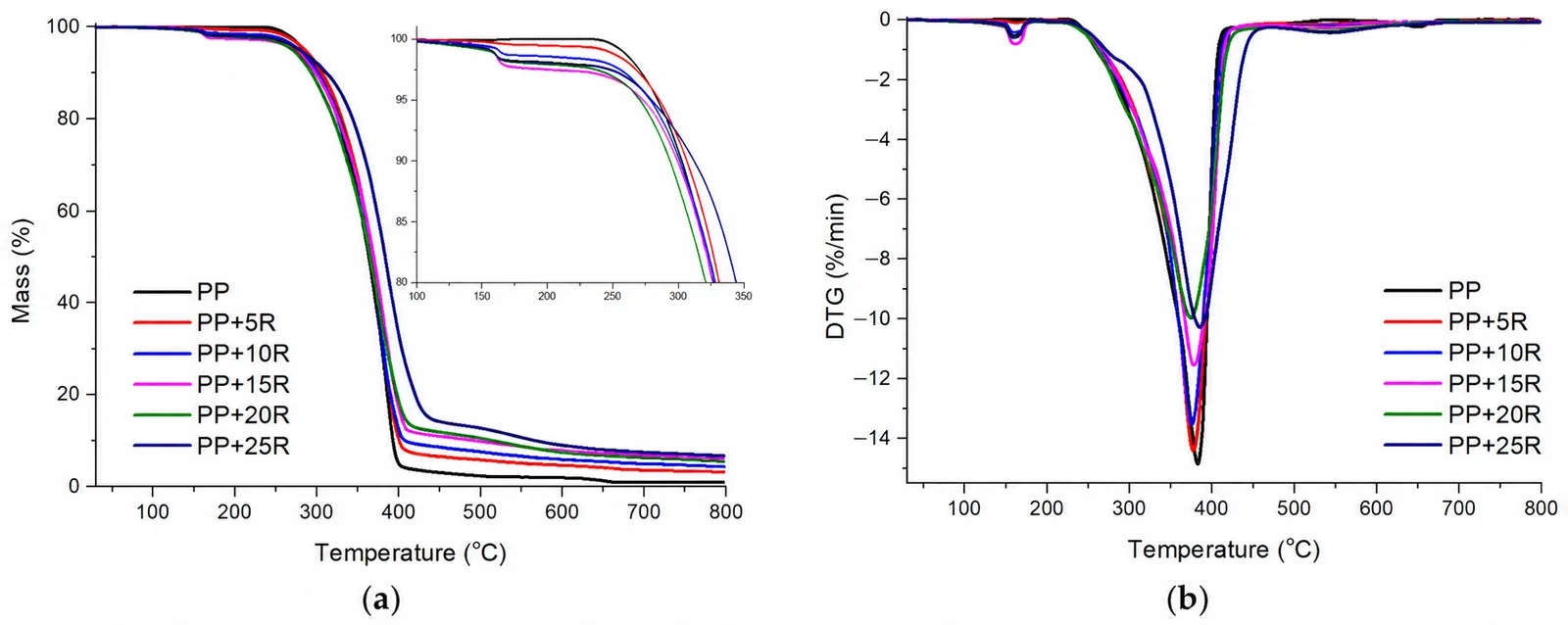
TGA (**a**) and DTG (**b**) curves of the PP-R composites after long-term NaCl absorption.

**Figure A3:**
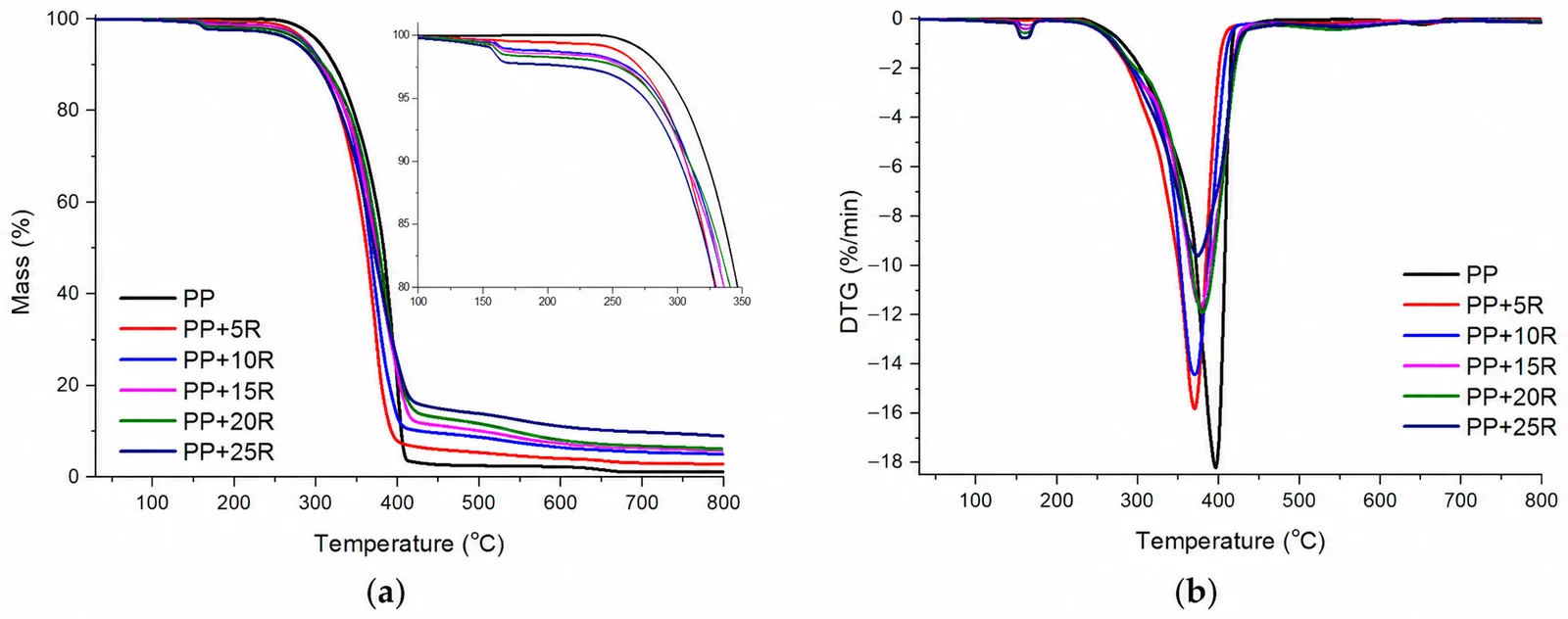
TGA (**a**) and DTG (**b**) curves of the PP-R composites after long-term H_2_SO_4_ absorption.

**Figure A4:**
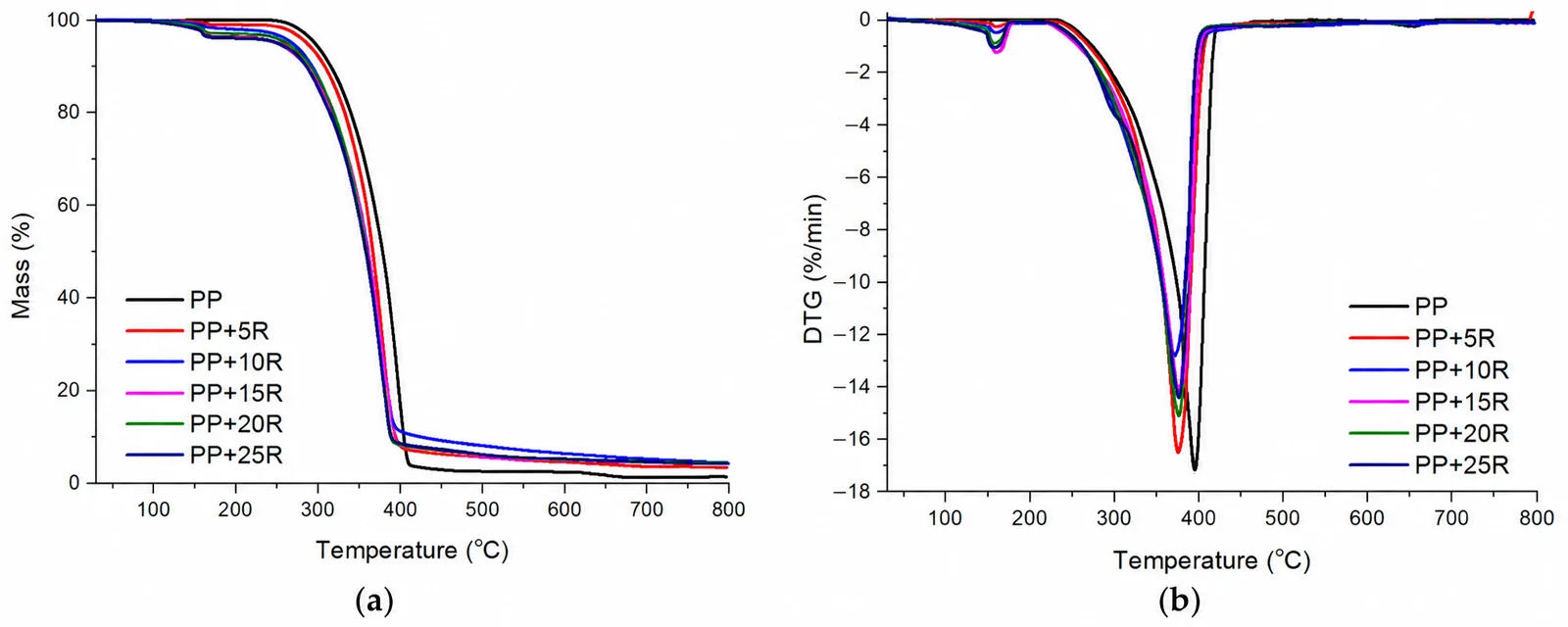
TGA (**a**) and DTG (**b**) curves of the PP-R composites after long-term NaOH absorption.

Thus, incubation in different environments brings only moderate changes in thermal stability, with minor differences in degradation temperatures based on the exposure environment, without compromising the fundamental thermal stability of the flame-retardant PP systems. The TGA/DTG curves together with some specific comments are presented in Appendix A, Figure A1, Figure A2, Figure A3 and Figure A4.

### 3.3. Dynamic Mechanical Analysis (DMA)

All initial PP-based systems exhibit a broad relaxation in the temperature range around −10 to 30 °C. In semicrystalline polymers such as PP, the glass transition temperature (Tg) of the amorphous phase is typically reported between −20 and 0 °C in dynamic mechanical measurements [39].

The damping factor (tan δ) peak is relatively weak and broad for all compositions, reflecting the semicrystalline nature of PP and the limited contribution of the amorphous phase to the overall viscoelastic response. In semicrystalline polypropylene, the glass transition of the amorphous phase is typically observed as a broad, weak relaxation in dynamic mechanical analysis rather than a sharp transition, due to constraints imposed by the crystalline regions, as demonstrated in classical dynamic mechanical studies [40,41].

**Figure 7:**
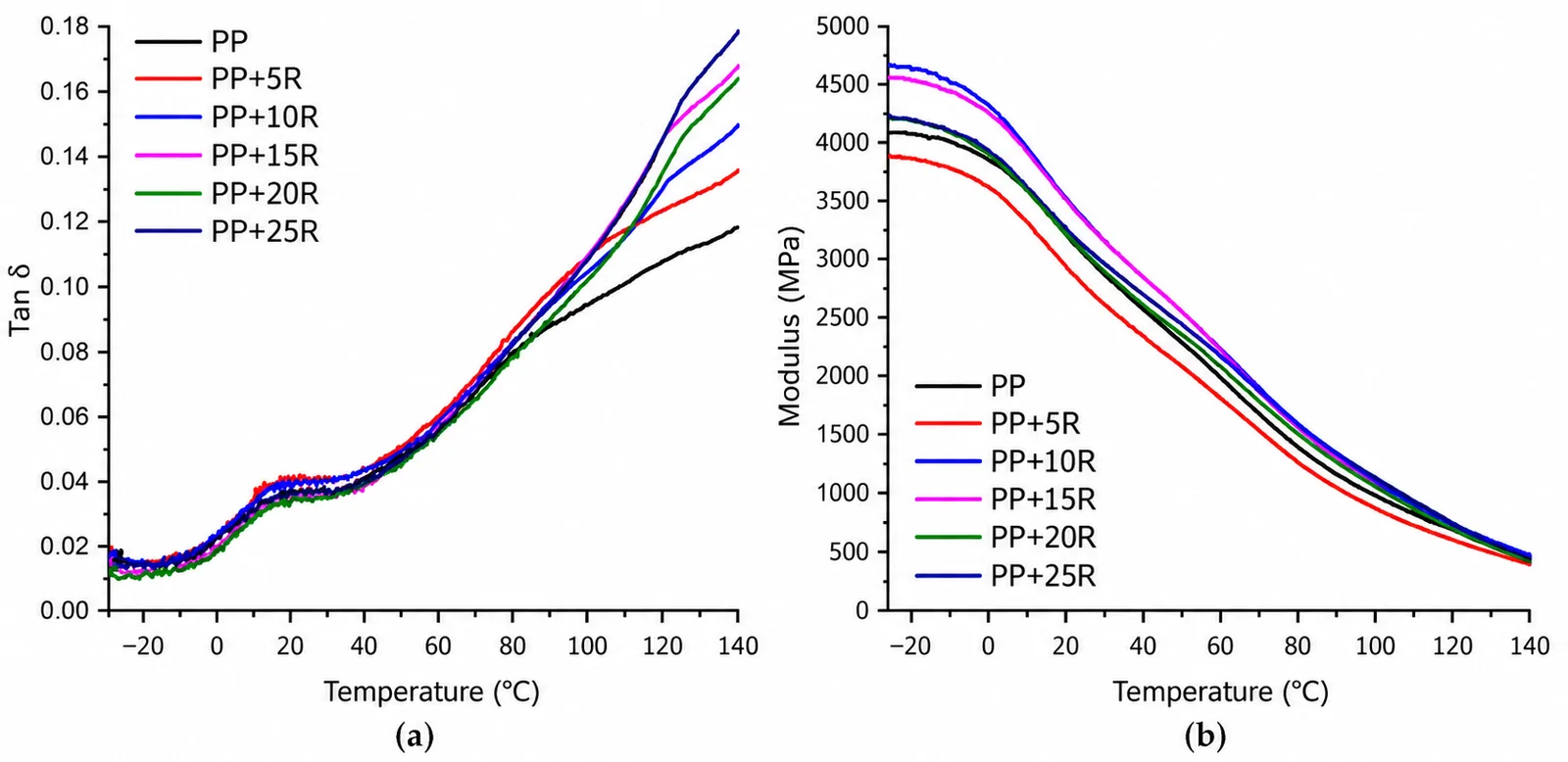
(**a**) Tan δ vs. temperature curves and (**b**) storage modulus (E′) as a function of temperature determined by DMA for the initial composites.

In this regard, the amorphous phase is not unique, but it is defined as free amorphous domains, with negative Tg (−20 …to 0 °C) and more relaxed constrained amorphous domains (interlamellar/rigid amorphous phase), with Tg at higher temperatures. These contributions overlap, and the relaxation of the constrained amorphous dominates tan δ, thus explaining this broad relaxation, with a maximum around 15–20 °C (Figure 7a), even though the thermodynamic Tg of the free amorphous is at negative temperatures.

No distinct additional relaxation peaks are detected upon incorporation of the flame-retardant additive, suggesting that the filler does not introduce new relaxation mechanisms but rather modulates the damping behavior of the PP matrix.

Regarding the storage modulus, E’ (Figure 7b), all systems exhibit values ranging from ~3800 MPa to ~4600 MPa, with a typical decrease with increasing temperature, reflecting the progressive softening of the PP matrix.

In the sub-ambient region (T < 0 °C), composites containing higher flame-retardant additive contents (10–25%) show higher E′ values compared with reference PP, indicating a clear stiffening contribution of the filler. It can be noted that PP+10%R and PP+15%R systems maintain the highest modulus levels across most of the investigated temperature range, suggesting improved resistance to thermal softening at intermediate additive contents.

Therefore, DMA curves show that the incorporation of the flame-retardant additive does not affect PP features but stabilizes the viscoelastic performance of PP across the investigated temperature range, both for the initial materials and after long-term exposure to corrosive environments, where the composite response remains qualitatively unchanged, with no additional relaxation peaks detected.

While environmental incubation induces moderate modifications of storage modulus, consistent with limited plasticization or mass variation observed in gravimetric measurements, the fundamental relaxation mechanisms of the PP matrix remain unchanged, as evidenced by the absence of a new tan δ relaxation peak.

**Figure A5:**
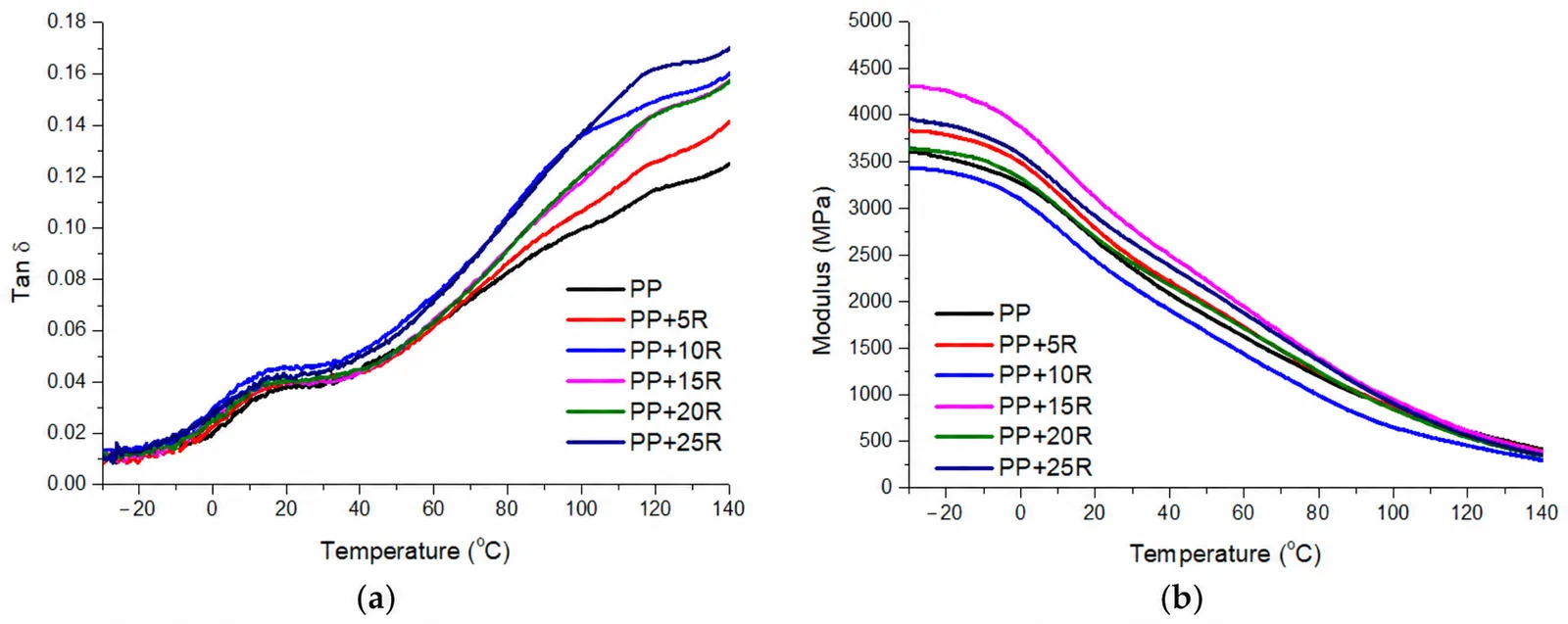
DMA results for the PP-R composites after water absorption: (**a**) tan δ vs. temperature curves and (**b**) storage modulus (E′) vs. temperature.

**Figure A6:**
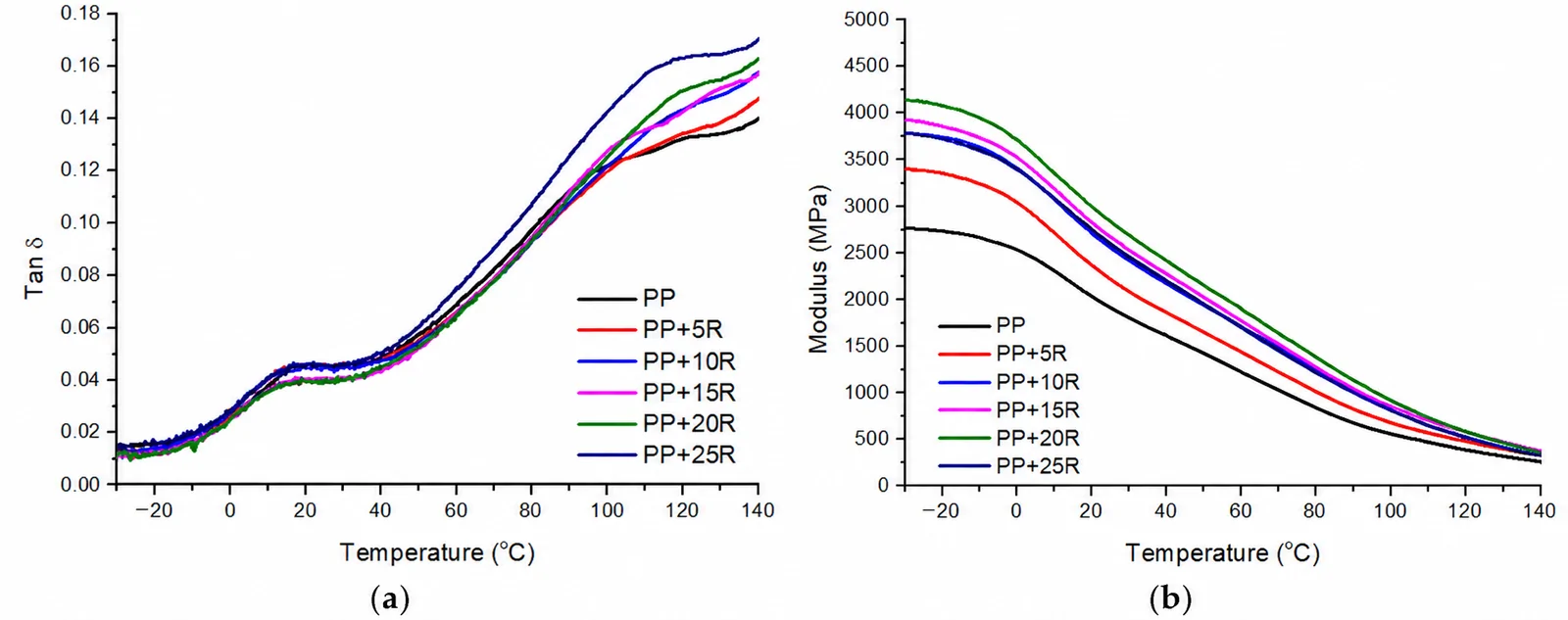
DMA results for the PP-R composites after NaCl incubation: (**a**) tan δ vs. temperature curves and (**b**) storage modulus (E′) vs. temperature.

**Figure A7:**
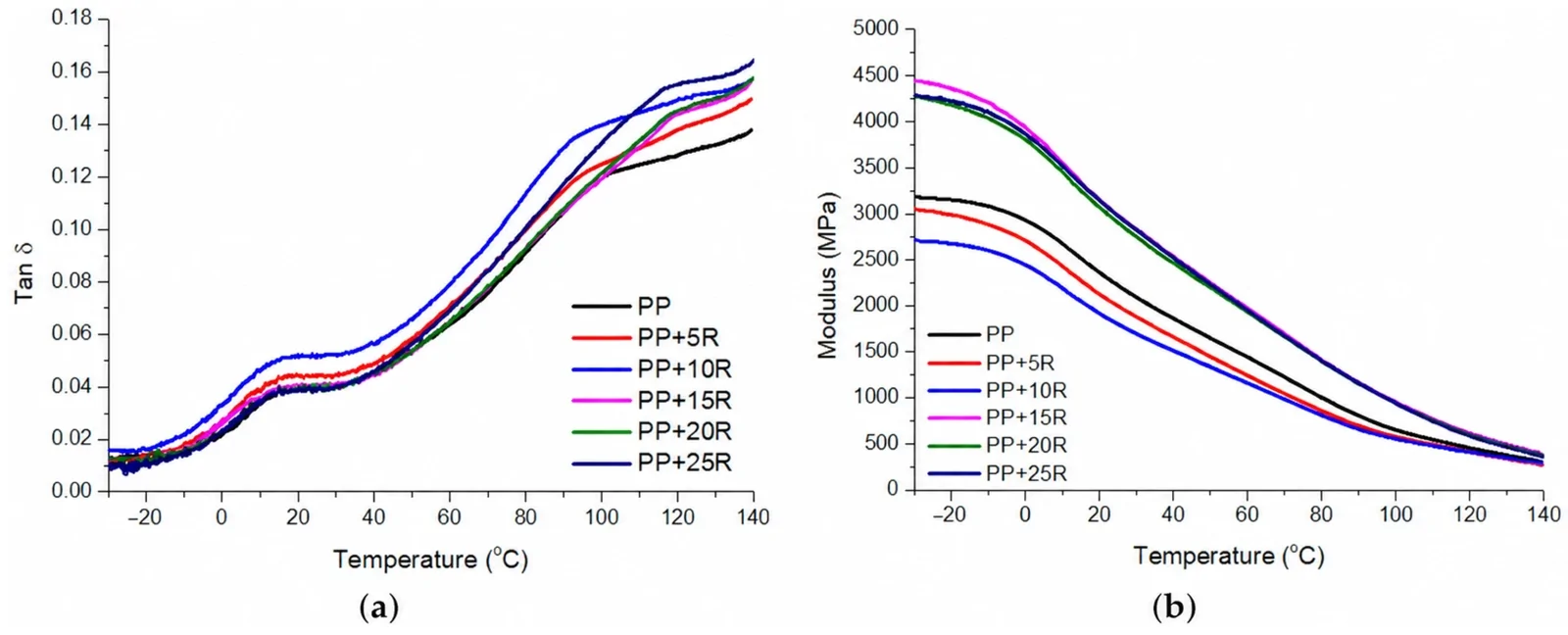
DMA results for the PP-R composites after H_2_SO_4_ incubation: (**a**) tan δ vs. temperature curves and (**b**) storage modulus (E′) vs. temperature.

**Figure A8:**
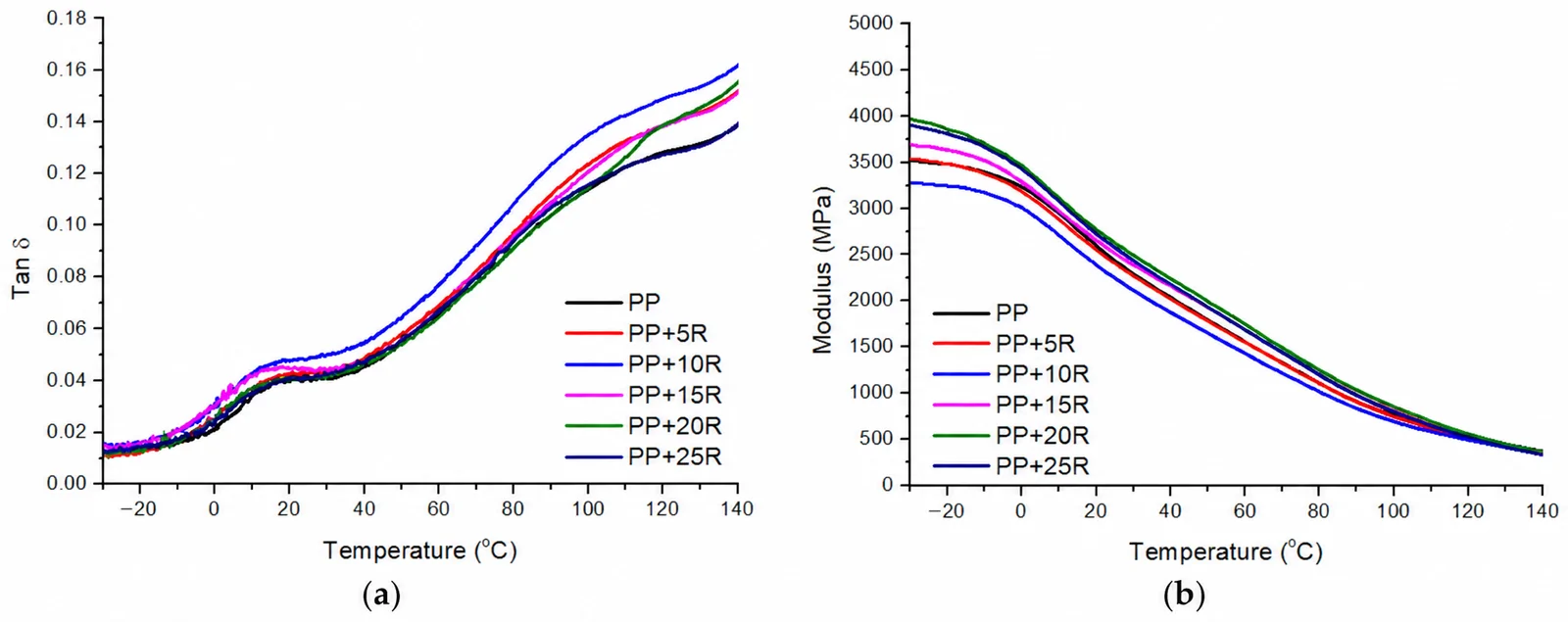
DMA results for the PP-R composites after NaOH incubation: (**a**) tan δ vs. temperature curves and (**b**) storage modulus (E′) vs. temperature.

These observations are consistent with the TGA results, which indicate single-step thermal degradation and comparable degradation temperatures after exposure, and also with the gravimetric measurements, confirming that the incorporated flame-retardant additive does not compromise the thermal or mechanical integrity of PP but rather contributes to maintaining stiffness and structural stability even under prolonged corrosive conditions. The DMA curves obtained after long-term exposure to the investigated media, together with additional comments regarding the viscoelastic response of the flame-retardant PP composites, are presented in Appendix B, Figure A5, Figure A6, Figure A7 and Figure A8.

## 4. Conclusions

The present study focused on the behavior of flame-retardant PP composites after long-term immersion in water, NaCl, H_2_SO_4_, and NaOH solutions. The experimental program considered a reference PP and PP-based systems containing 5, 10, 15, 20, and 25 wt.% of the phosphorus-based intumescent flame-retardant additive, in order to follow the influence of the additive content on the material response. The interpretation was based first on gravimetric measurements and then correlated with TGA/DTG and DMA results.

The gravimetric data showed that the reference PP had reduced interaction with the investigated media, consistent with the hydrophobic character of the polymer matrix. The additive-containing systems presented moderately higher absorption/degradation degree values, especially for the compositions with intermediate and high additive contents. The higher AD values observed for several additive-containing formulations indicate an increased gravimetric interaction with the immersion media compared with the neat PP. However, the PP-Reogard systems did not follow a consistent trend of progressive mass loss over the entire exposure period.

Dividing the immersion period into early, mid, and late exposure intervals allowed clearer interpretation of the experimental data. The early intervals described the initial contact between the specimens and the liquid media, while the mid and late intervals allowed the subsequent evolution of the gravimetric response to be evaluated. Isolated negative values or local variations observed at certain time points were interpreted as net gravimetric changes and were not, by themselves, considered evidence of chemical degradation.

The gravimetric response was dependent on both the immersion medium and the Reogard content. In the water and NaCl solution, the investigated systems exhibited predominantly positive A values, whereas exposure to acidic and alkaline media resulted in more variable, composition-dependent responses. Overall, the incorporation of Reogard modified the gravimetric response of PP to the investigated media. However, these gravimetric measurements do not allow the attribution of the observed mass variation to liquid uptake/retention, desorption, additive leaching, surface residue formation, or chemical degradation. Furthermore, the experiments were conducted under static/non-renewed immersion conditions. Consequently, possible changes in the chemistry of the exposure media during prolonged contact with the specimens may also have contributed to the observed gravimetric response.

The TGA/DTG analysis confirmed that the main degradation profile of the PP-based systems was preserved after long-term exposure. All investigated materials maintained the main thermal degradation profile, characteristic of polypropylene composites. The differences observed in the degradation temperature range were moderate and depended on both additive content and exposure medium. Therefore, immersion in the selected media did not generate a new thermal degradation mechanism for the flame-retardant PP systems.

The DMA results also confirmed that no additional relaxation process was observed for the PP-R composites after exposure. The broad relaxation behavior of PP was maintained, while the storage modulus values showed that the additive continued to contribute to the stiffness of the composites. Thus, even if the additive increased the interaction with the liquid media, the dynamic mechanical features determined by DMA were preserved under the investigated conditions.

Therefore, under the conditions considered in this study, the incorporated flame-retardant additive increased the interaction of PP-based composites with water, saline, acidic, and alkaline media, but this interaction was not translated into a clear degradation trend. The TGA/DTG and DMA results showed that the investigated composites preserved their main thermal degradation profile and the overall dynamic mechanical response after long-term exposure. Within the limits of the investigated properties, the results indicate that the material response is governed predominantly by interactions with the exposure media rather than by a progressive degradation process.

## Data Availability

The data supporting the findings of this study are included in the article and in the appendices. Additional information related to the experimental data can be made available from the corresponding author upon request.

## Appendix A. Additional TGA/DTG Results After Long-Term Exposure

After long-term water exposure, all PP-R systems indicate a single main degradation step (Figure A1a), meaning that immersion in water does not alter the fundamental thermal degradation mechanism of the polypropylene matrix. Only minor shifts in the onset of degradation are observed compared with the initial state, suggesting that water absorption induces limited physical changes rather than chemical degradation. The residual mass at high temperatures remains higher for additive-containing systems, confirming that the additive contribution to char formation is preserved after water immersion.

The DTG curves (Figure A1b) continue to display a single dominant degradation peak for all compositions, confirming the absence of additional degradation pathways induced by prolonged water exposure. A minor DTG signal observed at lower temperatures for all systems can be attributed to the evaporation of physically absorbed water remaining after long-term immersion.

It is important that the PP+5%R system exhibits a slightly lower DTG peak temperature compared with reference PP, indicating a modest shift toward earlier degradation at low additive content. Small filler additions can affect thermal degradation kinetics by altering local mobility and interface dynamics, which may manifest as minor shifts in TGA/DTG peak positions [38,42].

Slight variations in peak position and intensity are observed among the composites, with shifts to lower DTG peaks, which can be attributed to physical effects such as absorbed moisture release or microstructural rearrangements rather than polymer chain scission. The relative similarity of T_max_ values before and after exposure indicates that long-term water absorption does not significantly compromise the intrinsic thermal stability of the PP matrix.

After long-term NaCl exposure, all PP-R systems exhibit very similar TGA profiles (Figure A2a), with the main mass loss occurring within a narrow temperature range and no significant differences in thermal stability among the compositions.

Notably, the DTG maximum of all systems (Figure A2b) are centered near 400 °C after NaCl immersion, indicating a slight shift toward higher degradation temperatures compared to the initial state, while preserving a single-step degradation mechanism.

After long-term H_2_SO_4_ exposure, the TGA curves remain largely similar across all systems (Figure A3a), preserving a single main mass-loss step and indicating no major change in the overall thermal degradation profile.

The DTG curves show a single dominant degradation peak with comparable peak positions among the systems (Figure A3b), suggesting that H_2_SO4 immersion does not induce a pronounced shift in the maximum degradation temperature within the resolution of the presented data.

After long-term NaOH exposure, all PP-R composites display highly similar TGA profiles, with a single main mass-loss step and no evident changes in the overall thermal stability compared with the initial state (Figure A4a).

The DTG curves show a single dominant degradation peak at comparable temperatures for all compositions, indicating that prolonged NaOH immersion does not significantly affect the degradation kinetics of either reference PP or the additive-containing composites (Figure A4b).

## Appendix B. Additional DMA Results After Long-Term Exposure

After long-term water absorption, all systems preserve the same broad relaxation behavior observed in the initial composites, without observing any new relaxations (Figure A5), indicating that water uptake does not alter the fundamental viscoelastic properties of the studied PP-R composites.

The soft increase in tan δ intensity at higher temperatures for some additive-containing composites suggests enhanced damping, likely related to increased chain mobility in the amorphous regions promoted by water-filler interactions.

Almost all additive-modified composites retain higher E′ values than reference PP over most of the investigated temperature range. A general decrease in storage modulus compared with the initial state is observed for all systems, with E′ values remaining below 4000 MPa, except for PP+15%R, which reaches a little over 4300 MPa at low temperatures. These results indicate that water absorption does not compromise the stiffening efficiency of the filler, although it induces moderate modifications in the viscoelastic response of the composites.

After NaCl incubation, the tan δ curves retain the same broad relaxation profile as the initial materials, while the progressively higher tan δ values at elevated temperatures for additive-containing composites suggest increased damping without the appearance of additional relaxation processes (Figure A6a).

The storage modulus increases systematically with the additive content, indicating the preservation of the stiffening effect of the PP matrix with the addition of filler, when the composites are incubated long-term in a saline environment (Figure A6b).

At low temperatures, E′ rises from ~2750 MPa (PP) to ~3300 MPa (PP+5%R) and reaches ~3750–3800 MPa for PP+10–15%R and PP+25%R, with the highest value observed for PP+20%R (~4200 MPa), confirming the strongest reinforcement at intermediate-to-high additive loadings.

After H_2_SO_4_ incubation, tan δ curves maintain the same broad relaxation profile, with higher damping at elevated temperatures for additive-loaded composites, suggesting increased energy dissipation without the appearance of additional relaxation processes after acid exposure (Figure A7a).

The storage modulus response becomes clearly stratified into two distinct groups, indicating a strong composition-dependent effect of the acidic medium on viscoelastic stiffness (Figure A7b).

At low temperatures, the lowest E′ values are observed for PP+10%R (~2700 MPa) and PP+5%R (~3100 MPa), followed by reference PP (~3200 MPa), whereas the highest modulus levels are retained by composites with ≥15% additive content (PP+15%R ~4400 MPa; PP+20–25%R ~4300 MPa), evidencing pronounced reinforcement at intermediate-to-high filler contents. This behavior highlights the protective role of the flame-retardant additive, as higher filler contents (≥15%) enable the composites to retain significantly enhanced stiffness even after exposure to a highly corrosive medium such as H_2_SO_4_.

After NaOH incubation, tan δ curves preserve the same broad relaxation behavior observed in the initial composites, with no additional relaxation peaks, indicating that NaOH exposure does not alter the fundamental viscoelastic relaxation mechanisms of the PP matrix (Figure A8a).

A more important observation can be made for storage modulus, with E′ values increasing from ~3250 MPa (PP+10%R) to ~4000 MPa for PP+20%R, with none of the systems exceeding this value (Figure A8b).

Reference PP and PP+5%R exhibit similar modulus values (~3500 MPa), while higher additive contents (15–25%) progressively enhance stiffness, with PP+20%R showing the highest E′.

Thus, NaOH exposure indicates a moderate overall softening effect induced by the alkaline medium, without compromising the reinforcing role of the flame-retardant additive. This moderate decrease in storage modulus observed after NaOH exposure could be attributed to limited plasticization of the free amorphous phase, while the reinforcing effect of the additive remains effective through constrained amorphous regions and load transfer mechanisms, as commonly described for semicrystalline PP-based composites in dynamic mechanical studies.
